# Predicting the epidemic threshold of the susceptible-infected-recovered model

**DOI:** 10.1038/srep24676

**Published:** 2016-04-19

**Authors:** Wei Wang, Quan-Hui Liu, Lin-Feng Zhong, Ming Tang, Hui Gao, H. Eugene Stanley

**Affiliations:** 1 Web Sciences Center, University of Electronic Science and Technology of China, Chengdu 610054, China; 2 Big data research center, University of Electronic Science and Technology of China, Chengdu 610054, China; 3 Center for Polymer Studies and Department of Physics, Boston University, Boston, Massachusetts 02215, USA

## Abstract

Researchers have developed several theoretical methods for predicting epidemic thresholds, including the mean-field like (MFL) method, the quenched mean-field (QMF) method, and the dynamical message passing (DMP) method. When these methods are applied to predict epidemic threshold they often produce differing results and their relative levels of accuracy are still unknown. We systematically analyze these two issues—relationships among differing results and levels of accuracy—by studying the susceptible-infected-recovered (SIR) model on uncorrelated configuration networks and a group of 56 real-world networks. In uncorrelated configuration networks the MFL and DMP methods yield identical predictions that are larger and more accurate than the prediction generated by the QMF method. As for the 56 real-world networks, the epidemic threshold obtained by the DMP method is more likely to reach the accurate epidemic threshold because it incorporates full network topology information and some dynamical correlations. We find that in most of the networks with positive degree-degree correlations, an eigenvector localized on the high *k*-core nodes, or a high level of clustering, the epidemic threshold predicted by the MFL method, which uses the degree distribution as the only input information, performs better than the other two methods.

Because many real-world phenomena incorporate spreading dynamics on complex networks, the topic has received much attention over the last decade^1^,^2^. Notable examples include the spread of sexually-transmitted diseases through contact networks^3^, the spread of malware on wireless networks^4^, and the spread of computer viruses through email networks^5^. In each case the spreading dynamics are strongly affected by network topology, and this complicates the task of understanding their behavior. Existing studies of spreading dynamics have focused on both theoretical aspects (e.g., nonequilibrium critical phenomena^6^,^7^) and practical issues (e.g., proposing efficient immunization strategies^8^,^9^). Researchers have focused on developing ways of accurately identifying epidemic thresholds because of their important ramifications in many real-world scenarios. Theoretically speaking, an epidemic threshold characterizes the critical condition above which a global epidemic occurs^7^. Being able to predict an epidemic threshold allows us to determine the critical exponents^10^ and Griffiths effects^11^, which are important in research on nonequilibrium phenomena^6^. Practically speaking, quantifying an epidemic threshold allows us to determine the effectiveness of a given immunization strategy^8^. A proposed immunization strategy is effective if it increases the epidemic threshold. In addition, knowing the epidemic threshold enables us to more accurately determine the optimum source node^12^.

Researchers have put much effort into developing a theory for quantifying the thresholds in epidemic spreading models such as the susceptible-infected-recovered (SIR) model^1^. The best-known theoretical methods fall into three categories based on the topology information that they use. The first is the mean-field like (MFL) approach, which uses the degree distribution as the sole input parameter. This category includes the heterogeneous mean-field theory^7^,^13^, the percolation theory^14^, the edge-based compartmental approach^15^,^16^,^17^,^18^, and the pairwise approximation method^19^,^20^. The second type is the quenched mean-field (QMF) method that describes network topology in terms of the adjacent matrix. Examples include the discrete-time Markov chain^21^ and the *N*-intertwined approach^22^. The third type is the dynamical message passing (DMP) method^23^ that describes network topology in terms of the non-backtracking matrix. This approach is accurate in the case of tree-like networks. Researchers have used these three approaches to uncover the macroscopic statistical characteristics (e.g., degree^7^ and weight distributions^17^), mesoscale structure (e.g., degree-degree correlations^24^, clustering^25^ and community^26^), and microcosmic characteristics (e.g., node degree^27^ and edge weight^17^) that strongly affect the epidemic threshold. For example, uncorrelated or correlated networks with a strongly heterogeneous degree distribution can, under certain conditions, reduce or even eliminate the epidemic threshold^7^,^24^.

The theoretical approaches always assume (i) that an epidemic can spread on a large, sparse network^7^,^14^,^16^,^28^, (ii) that dynamical correlations among the neighbors do not exist^7^, and (iii) that all the nodes or edges within a given class are statistically equivalent^7^,^17^. These three methods also usually focus on a class of networks, such as uncorrelated networks, clustering networks, and community networks. In any given network, the three theoretical methods usually predict different epidemic thresholds^29^. To determine the relationships among the three differing outcomes of the MFL method, the QMF method, and the DMP method and to determine which more closely describes real-world epidemic thresholds, we use a comprehensive study of the SIR model on uncorrelated configuration networks and of a group of 56 real-world networks. We find that the MFL and DMP methods predict the same epidemic threshold value for uncorrelated configuration networks and that this value is larger and more accurate than the value predicted by the QMF method. The relationships among the three theoretical predictions for real-world networks, however, remain unclear. In the 56 real-world networks studied, the DMP method performs the best in most cases because it considers the full topology and many of the dynamical correlations among the states of the neighbors, but due to the localized eigenvector of the adjacent matrix the QMF method often deviates from accurate epidemic threshold values. For networks with an eigenvector localized on the high *k*-core nodes, positive degree-degree correlations, or high clustering, the prediction by MFL method is more likely to be accurate than the predictions from other two methods, even though the MFL method uses the degree distribution as the sole input parameter. For networks with an eigenvector localized on the hubs, negative degree-degree correlations, or low clustering, the DMP method performs the best in most occasions. Finally, we note that the performances of the three predictions do not exhibit an obvious regularity versus the modularity, and in most cases the DMP method performs better than other two.

## Results

### Theoretical predictions of epidemic threshold

In the SIR pattern of the spread of disease through a network, at any given time each node is either susceptible, infected, or recovered. A susceptible node does not transmit the disease. Infected nodes contract the disease and spread it to their neighbors. A recovered node has returned to health and no longer spreads the disease. The synchronous updating method^30^ is applied to renew the states of nodes. To initiate the epidemic, we randomly select a “seed” node and designate all other nodes susceptible. At each time step, infected nodes transmit the disease to susceptible neighbors with a probability *β*. Infected nodes can also recover with a probability *γ*. The spreading terminates when all infected nodes have recovered. The spreading dynamics can be characterized by the effective spreading rate *λ* = *β*/*γ*. More details are shown in the Supporting Information. When *λ* is below the epidemic threshold *λ*_*c*_ (i.e., *λ* ≤ *λ*_*c*_), the disease spreads locally (i.e., only a tiny fraction of nodes transmit the disease). Epidemics can occur when *λ* > *λ*_*c*_ (i.e., when a finite fraction of nodes transmit the disease).

The mean-field like (MFL) method, the quenched mean-field (QMF) method, and the dynamical message passing (DMP) method are commonly-used theoretical methods of predicting an epidemic threshold. In this section we clarify the relationships among these epidemic thresholds predicted by the three theoretical methods.

The mean-field like (MFL) method incorporates the heterogeneous mean-field theory, percolation theory, the edge-based compartmental approach, and the pairwise approximation method. Here the epidemic threshold is predicted by using only the degree distribution, and it is assumed that (i) all the nodes and edges in a given class are statistically equivalent, (ii) the states of nodes among neighbors are independent, and (iii) the network size is infinite. Using the degree distribution *P*(*k*) as the only input parameter, the theoretical epidemic threshold prediction using the MFL method is


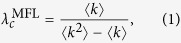


where 〈*k*〉 and 〈*k*^2^〉 are the first and second moments of the degree distribution, respectively. Although 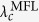 is a good predictor of the epidemic threshold in uncorrelated networks, the prediction may fail in real-world networks because of their complex structure (e.g., degree-degree correlations, clustering, and community) and the strong dynamical correlations among the states of neighbors^27^,^31^.

The quenched mean-field (QMF) method^21^,^32^,^33^ takes into account the complete network structure by using the adjacent matrix *A*. This distinguishes it from the MFL method, which simply uses the degree distribution. The adjacent matrix *A* is also used to describe network topology by the discrete-time Markov chain^21^, the *N*-intertwined method^22^, and other similar methods, and thus they fall into the same class as the QMF method. The QMF method is unable to capture the dynamical correlations among the states of neighbors and uses only the correlation between the theoretical epidemic threshold and the leading eigenvalue of the adjacent matrix to predict the epidemic threshold, i.e.,


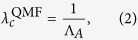


where the leading eigenvalue of the adjacent matrix is^22^


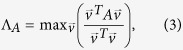


where 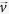 is a column vector with *N* elements, and *N* is the network size. Note that the epidemic threshold predicted by Eq. (2) is the same with the lower bound of epidemic threshold of SIS model^33^. Since the epidemic threshold of SIS model is smaller than that of SIR model^34^, we know that 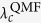 is precise a lower bound of epidemic threshold of SIR model.

The dynamical message passing (DMP) method was recently developed and used to study nonreversible epidemic spreading dynamics in an SIR modeled finite-sized network^23^,^28^,^35^. The DMP method uses the non-backtracking matrix to determine the complete network structure. This method can both describe the complete network structure and capture some of the dynamical correlations among the states of neighbors that are neglected in the MFL and QMF methods. In large sparse networks the DMP method provides a good estimation of the epidemic threshold, i.e.,


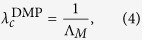


where


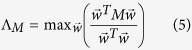


is the leading eigenvalue of the non-backtracking matrix^36^,^37^,^38^,^39^


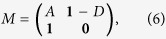


and **1** is a *N* × *N* unit matrix, *D* is the diagonal matrix with the vertex degrees along its diagonal, and **0** is a *N* × *N* null matrix. From Eqs (1, 2 and 4), we know that the predicted epidemic threshold of SIR model has the same formula with the bond percolation model^36^. Since the SIR spreading is a dynamical evolution process, the interplay between complex structures and dynamical correlations may result in a distinct accurate critical point from the bond percolation model^40^,^41^. Therefore, how the above three classical theoretical methods perform in predicting the epidemic threshold of SIR model in complex networks is worth pursuing.

The three theoretical predictions of epidemic threshold are closely correlated. In any given network they distinct, e.g., 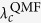 is less than 〈*k*〉/〈*k*^2^〉^12^. To determine other relationships among the three theoretical thresholds, we assume that *κ* is a eigenvalue of non-backtracking matrix *M* and that 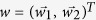 is the corresponding eigenvector of *κ*, where 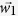 and 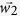 are the first and last *N* elements of vector *w*, respectively. Using Eq. (6), the eigenvalue problem is written


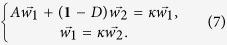


Multiplying the left vector 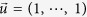 on the first line of (7) and combining the second line of (7) yields


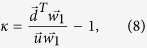


where 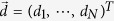 and *d*_*i*_ is the degree of node *i*. In uncorrelated networks the nonbacktracking centrality of a node is proportional to its degree^37^, i.e., 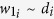. Here the theoretical prediction 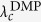 using the DMP method is the same as 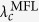 using the MFL method.

To examine the eigenvalue relationships between the adjacent matrix and non-backtracking matrix, we insert the second equation of (7) into the first equation and obtain


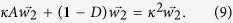


Multiplying 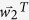 on both sides of Eq. (9) and dividing 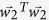, we get


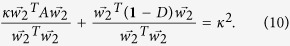


Using matrix theory^22^ we know that the eigenvalue 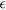 and its corresponding eigenvector 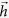 of a matrix 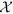 satisfy 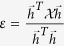. We assume that *ξ*_1_ and *ξ*_2_ are the eigenvalue of *A* and **1** − *D*, respectively, i.e., 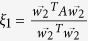 and 
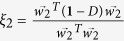
. Thus Eq. (10) can be written as


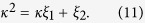


Because the minimum eigenvalue of **1** − *D* is 1 − *k*_max_, we find that


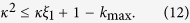


Rewriting Eq. (12) we get


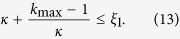


Note that *κ* and *ξ*_1_ are the eigenvalues of matrixes *M* and *A* respectively, and we get


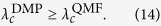


With similar arguments in ref. 42 and combining Eq. (14), we know that 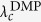 is a tight lower bound of the accurate epidemic threshold *λ*_*c*_ for local tree-like networks. For real-world networks, the basic assumption (i.e., local tree-like) can not always be satisfied, thus, 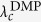 is possible larger than *λ*_*c*_.

Many real-world networks have a heterogeneous degree distribution, e.g., a power-law degree distribution 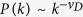, where *ν*_*D*_ is the degree exponent. In uncorrelated scale-free networks, 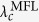 vanishes in the thermodynamic limit when *ν*_*D*_ < 3 because 〈*k*^2^〉 diverges. When *ν*_*D*_ > 3, 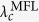 is a finite value. Using the QMF method, the epidemic threshold 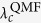 is determined by the maximum degree *k*_max_. When the degree exponent *ν*_*D*_ > 2.5, and 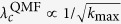. When *ν*_*D*_ < 2.5, we have 
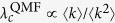
43, which indicates that 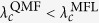. Note that 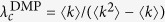 for uncorrelated networks^38^ is the same with 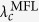. According to Eq. (14), 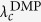 is always larger than 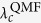. Unfortunately, the complex topology of the real-world networks makes the relationships among the three types of prediction unclear.

### Simulation results

Increasing the amount of network topology information utilized in any predictive method, the intuitional understanding tells us that the better performance of the method. Using the assumptions listed in previous section, we expect the DMP method to outperform the QMF method and the QMF method to outperform the MFL method. We next evaluate the performance of the three types of method using a large number on SIR studies of (i) uncorrelated configuration networks, and (ii) 56 real-world networks. We employ the estimators supplied in previous section to determine the theoretical epidemic threshold, and use the relative variance to determine the accurate epidemic threshold (see details in Method).

To better understand the performance of the three types of method, we further classify the networks into two classes according to the distinct eigenvector localizations of the leading eigenvalue of the adjacent matrix^44^, i.e., (i) localized hub networks (LHNs) in which the leading eigenvalue of the adjacent matrix Λ_*A*_ is closer to 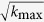 than 〈*k*^2^〉/〈*k*〉, where *k*_max_ is the maximum degree of the network (the eigenvector is localized on the hub nodes), and (ii) localized *k*-core networks (LKNs) in which Λ_*A*_ is closer to 〈*k*^2^〉/〈*k*〉 than 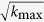 (the eigenvector is localized on nodes with a high *k*-core index).

### Uncorrelated configuration networks

**Figure 1:**
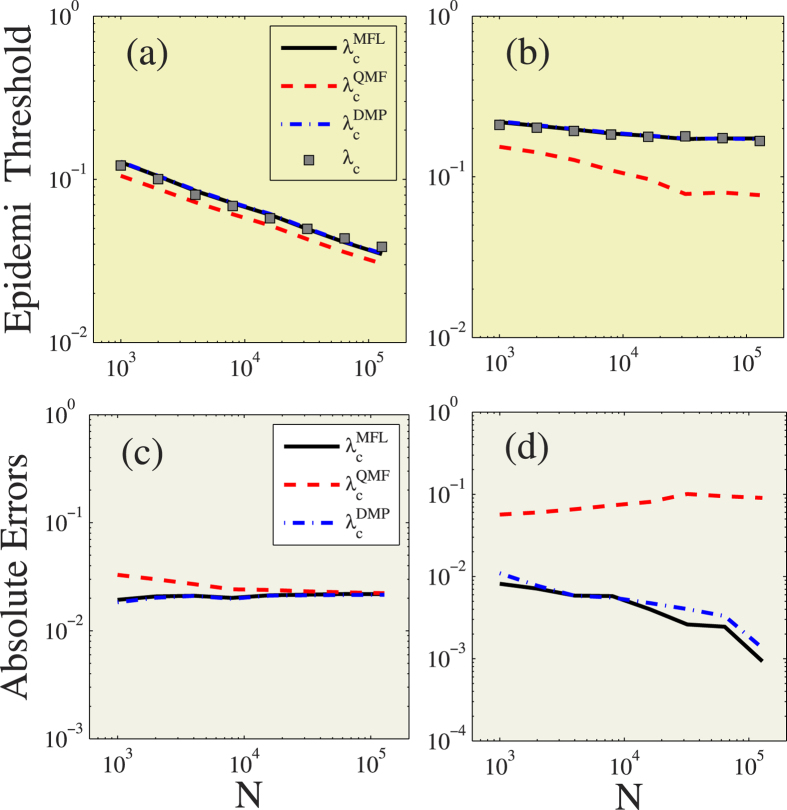
Predicting epidemic threshold for uncorrelated configuration networks under different network sizes. Theoretical predictions of 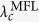 (black solid lines), 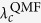 (red dashed lines), 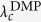 (blue dash-dotted lines) and numerical prediction (gray squares) versus network size *N* for degree exponent *ν*_*D*_ = 2.1 (**a**) and *ν*_*D*_ = 3.5 (**b**). The absolute errors between *λ*_*c*_ and 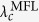 (black solid lines), 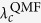 (red dashed lines) and 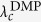 (blue dash-dotted lines) versus *N* for *ν*_*D*_ = 2.1 (**c**) and *ν*_*D*_ = 3.5 (**d**).

Figure 1 shows a systematic study of the SIR model on uncorrelated configuration networks. We focus on size *N* scale-free networks with power-law degree distributions, i.e., 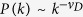, where *ν*_*D*_ is the degree exponent. The minimum degree is *k*_min_ = 3, and the maximum degree *k*_max_ is set at 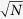, which ensures that there will be no degree-degree correlations in the thermodynamic limit. Without lack of generality, we can set *γ* = 1 in simulations. Two values, *ν*_*D*_ = 2.1 and *ν*_*D*_ = 3.5, are considered. According to definition^44^, networks with *ν*_*D*_ = 2.1 are LKNs and networks with *ν*_*D*_ = 3.5 are LHNs. Figure 1 shows that predictions from the MFL (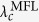) and DMP (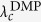) methods in general produce similar theoretical values and perform better than the prediction from the QMF (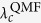) method. When *ν*_*D*_ = 2.1, the absolute errors in the epidemic threshold from the MFL and DMP methods are very small for all values of *N*, and the absolute errors from the QMF method decrease with *N*. The absolute error for method *u* ∈ {MFL, QMF, DMP} is 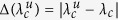. When *ν*_*D*_ = 3.5, the absolute error from the QMF method stabilizes to finite values even in infinitely large networks, and the absolute errors for the MFL and DMP methods decrease with *N*. From these results we find that the performance of the QMF method is counterintuitive, i.e., that its performance is even worse than the MFL method. At the same time, all of these results confirm the relationships among the three theoretical predictions for uncorrelated networks previously discussed.

### Real-world networks

We now examine the performances of the three theoretical predictions 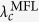, 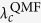 and 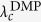 on a group of 56 real-world networks of various types, e.g., social networks, citation networks, infrastructure networks, computer networks, and metabolic networks. The Supporting Information supplies additional statistical information about these real-world networks. Note that spreading processes are performed on giant connected clusters. At times, for the sake of simplicity, we treat the directed networks as undirected and the weighted networks as unweighted.

**Figure 2:**
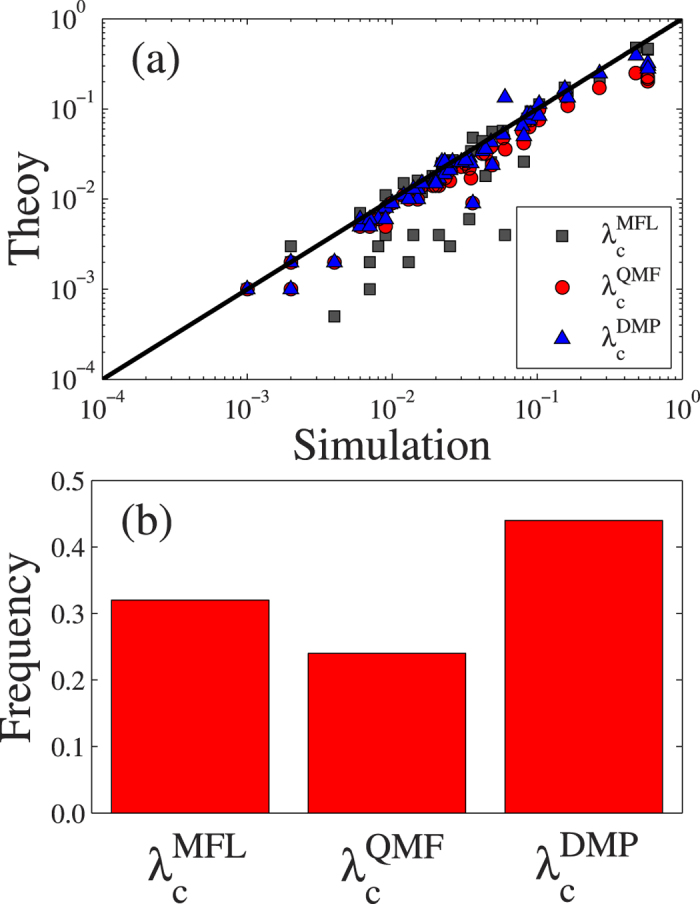
Comparing the accuracy between three types of theoretical and numerical predictions of the epidemic threshold on 56 real-world networks. (**a**) Theoretical predictions of 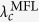 (gray squares), 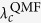 (red circles) and 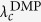 (blue up triangles) versus numerical predictions *λ*_*c*_ of the epidemic threshold. (**b**) In all the entire sample of real-world networks, the fraction of 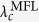 [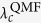 or 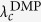] is the closest value to *λ*_*c*_.

Figure 2(a) shows the accuracy of 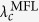, 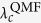, and 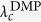 when applied to the 56 networks. Each symbol marks a theoretical prediction versus a numerical network prediction. We compute the relative frequency of 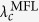, 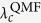, and 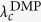 to determine which one produces a value closest to *λ*_*c*_ [see Fig. 2(b)]. Because the DMP method considers the full information of network topology and also some dynamical correlations, 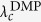 is the best prediction in more than 40% of the networks. The 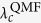 value is the closest to the actual epidemic threshold in 25% of the networks, and the epidemic threshold predicted by the MFL method, which uses the degree distribution as the only input parameter, is closest to the real epidemic threshold in about one-third of the real-world networks. Comparing these three predictions we find that the DMP method outperforms the other two, i.e., when determining the epidemic threshold in a general network, the DMP method is more frequently accurate than the other two.

**Figure 3:**
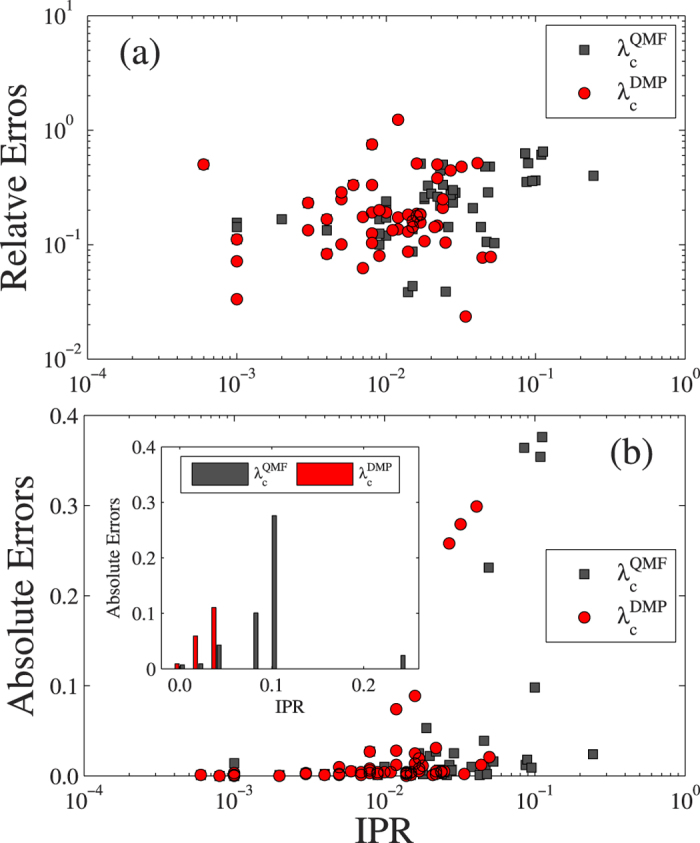
The effects of inverse participation ratio (IPR) of the adjacency and the nonbacktracking matrices on the accuracy of theoretical predictions. (**a**) The relative errors and (**b**) absolute errors as a function of IPR of the principal eigenvectors of the adjacency (black squares) and the nonbacktracking matrices (red circles). The inset of (**b**) is the average absolute errors as a function of IPR.

Theoretical predictions 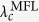 given by the MFL method often fail because it neglects much structural information and also all dynamical correlations. The performance of the QMF method is counterintuitive because of the localized eigenvector of the leading eigenvalue of the adjacent matrix [see Fig. 3(a)]. Figure 3 shows the effects of the inverse participation ratios (IPR)^39^,^45^ of the adjacent and non-backtracking matrixes. We find that the relative and absolute errors between the theoretical and numerical predictions increase with IPR, i.e., the QMF and DMP methods deviate from the accurate epidemic threshold more easily when IPR is large because the eigenvector centralities of adjacent and non-backtracking matrixes are localized on hub nodes or high *k*-core index nodes^44^. The relative error of method *u* ∈ {MFL, QMF, DMP} can be 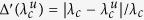.

**Figure 4:**
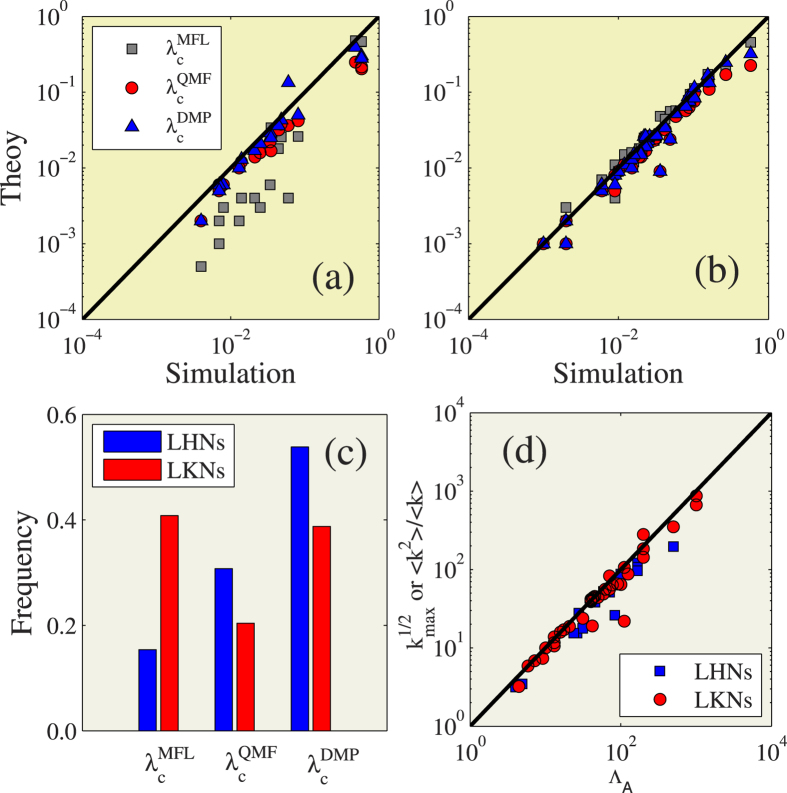
Verify the accuracy for three types of theoretical epidemic threshold on real-world networks. The theoretical predictions of 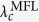 (gray squares), 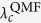 (red circles) and 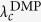 (blue up triangles) versus numerical predictions *λ*_*c*_ of the epidemic threshold on (**a**) LHNs and (**b**) LKNs. (**c**) In the collective of LHNs and LKNs of real-world networks, the fraction of 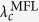 [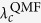 or 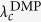] is the closest value to *λ*_*c*_. (**d**) The values of 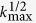 for LHNs and 〈*k*^2^〉/〈*k*〉 for LKNs versus the leading eigenvalue Λ_*A*_ of the adjacent matrix.

Recent research results indicate that networks have distinct eigenvector localizations^44^. In real-world networks they are either localized on hubs networks (LHNs) or localized on *k*-core networks (LKNs). Depending on the localization of the eigenvector of adjacent matrix, there are 19 LHNs and 37 LKNs among the 56 real-world networks. Figure 4(d) shows that the values Λ_*A*_ of LHNs are close to 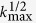 (blue squares), and the values Λ_*A*_ of LKNs are close to 〈*k*^2^〉/〈*k*〉 (red circles). In LHNs [see Fig. 4(a,c)] the three methods perform as we would expect. The DMP method is the best predictor and the MFL method the worst because it neglects much detailed network structure information. In contrast, in the LKNs [see Fig. 4(b,c)], the simple MFL method performs the best, and it is slightly accurate than the DMP method.

**Figure 5:**
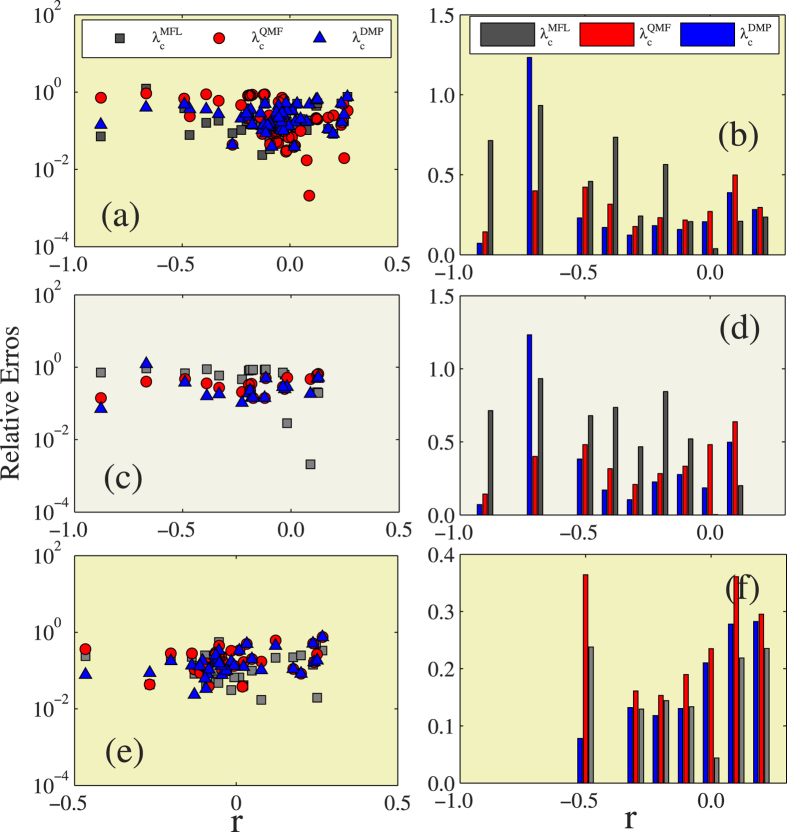
Effects of degree-degree correlations on the relative errors of different theoretical predictions. In the first column, figures (**a**,**c**,**e**) are the the relative errors of the three different theoretical predictions versus degree-degree correlations *r*. In the second column, figures (**b**,**d**,**f**) are the the average relative errors for the three different theoretical predictions versus *r*. The first row exhibits the results of 56 real-world networks, the second row shows the results of LHNs, the third row performs the results of the LKNs.

We now compare the accuracy between the three theoretical epidemic thresholds under different microscopic and mesoscale topologies of real-world structures, including degree-degree correlations *r*, clustering *c*, and modularity *Q*. To measure the accuracy of the three methods in each theoretical prediction, we compute the average relative errors in the interval (*x* − Δ*x*/2, *x* + Δ*x*/2), where *x* is *r*, *c*, and *Q*. Here we set Δ*x* = 0.1 unless otherwise specified. Figure 5(a,b) show that in all cases except the Facebook (NIPS) network the DMP method has a lower relative error when the Pearson correlation coefficient value is *r* < 0. The Facebook (NIPS) network may be an exception because the IPR value of its non-backtracking matrix is relatively large, i.e., 0.012. When *r* < 0, we can conclude that the DMP method performs the best and the MFL method performs the worst. When *r* > 0, the MFL method is the most accurate and the QMF method is the least. Figure 5(c–f) show the 56 real-world networks, separating them according to eigenvector localization. In LHNs we see a phenomenon similar to that shown in Fig. 5(a,b), i.e., when *r* < 0 the DMP method is the most accurate and the MFL method is the least, but when *r* > 0 the MFL method is the most accurate and the QMF method is the least. In LKNs, when *r* < 0 the DMP method is the most accurate, when *r* > 0 the MFL method is the most accurate, and the QMF method is always the least accurate. This suggests that the MFL method is the best for predicting epidemic thresholds in networks with positive degree-degree correlations, but that the DMP method is better in all other cases.

**Figure 6:**
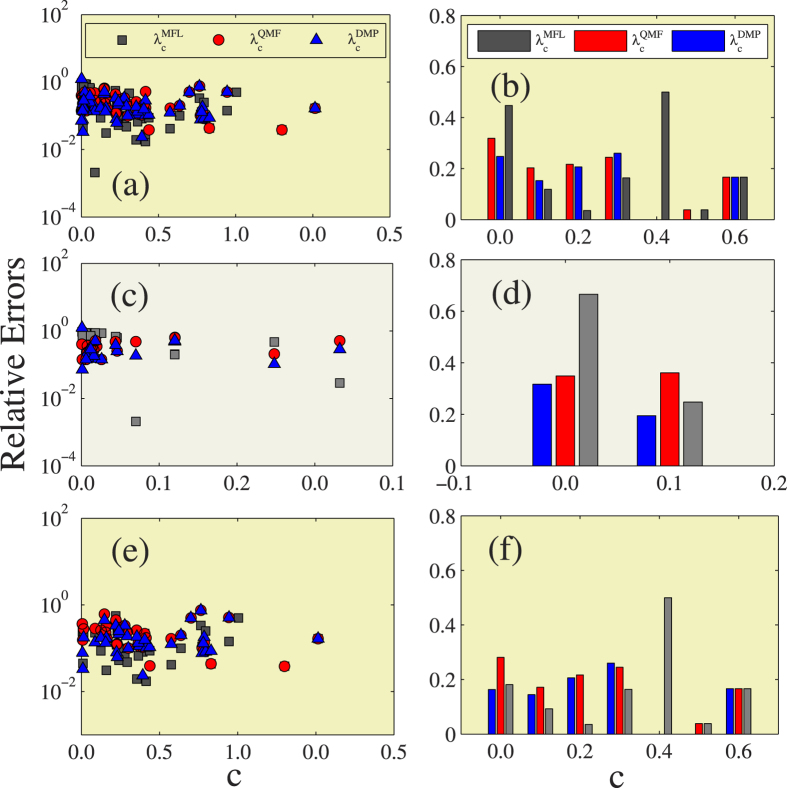
Effects of clustering on the relative errors of different theoretical prediction. In the first column, figures (**a,c,e**) are the the relative errors of the three different theoretical predictions versus clustering *c*. In the second column, figures (**b,d,f**) are the the average relative errors for the three different theoretical predictions versus *c*. The first row exhibits the results of 56 real-world networks, the second row shows the results of LHNs, the third row performs the results of the LKNs.

Using an analytic framework similar to that shown in Fig. 5, we compare the accuracy among the three theoretical predictions under different clustering coefficient *c* in Fig. 6. Figure 6(a,b) show that when *c* < 0.1, the relative error of the DMP method is the lowest and the relative error of the MFL method is the largest. When *c* > 0.1, the relative error of the MFL method is the lowest and the relative error of the QMF method is, in most cases, the largest. Thus when *c* < 0.1 the DMP method is the most accurate in predicting the epidemic threshold, but when *c* > 0.1 the MFL method is the most accurate. In LHNs, we find the same phenomena as shown in Fig. 6(a,b). The DMP method is the best predictor when *c* < 0.1, and the MFL method the best when *c* > 0.1 [see Fig. 6(c,d)]. Figure 6(e,f) show that in LKNs the DMP method performs the best for small *c* and the MFL method the best for large *c*.

**Figure 7:**
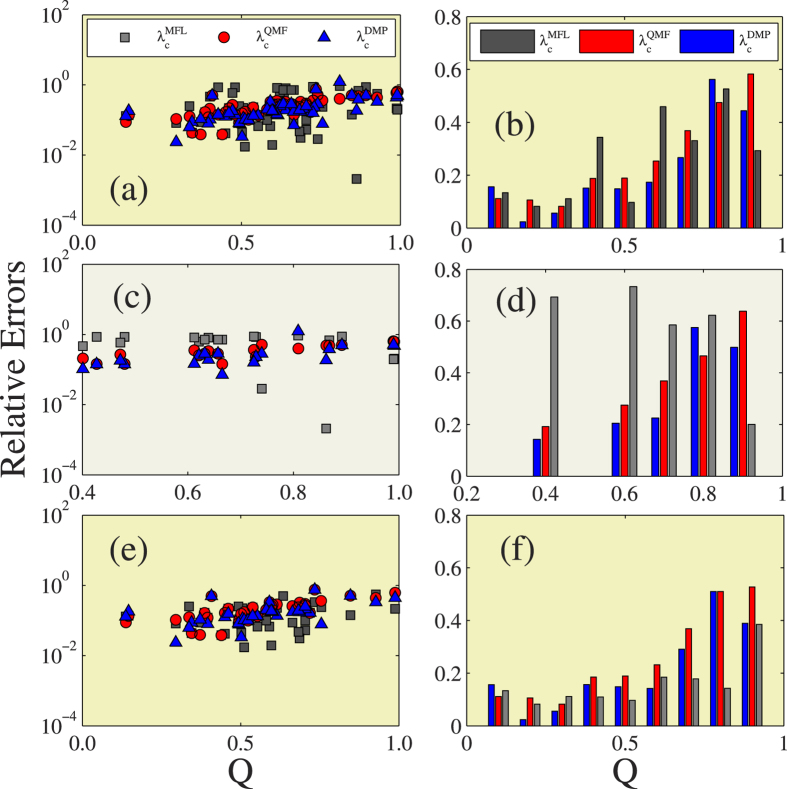
Effects of modularity on the relative errors of different theoretical prediction. In the first column, figures (**a,c,e**) are the the relative errors of the three different theoretical predictions versus modularity *Q*. In the second column, figures (**b,d,f**) are the the average relative errors for the three different theoretical predictions versus *Q*. The first row exhibits the results of 56 real-world networks, the second row shows the results of LHNs, the third row performs the results of the LKNs.

Finally, Fig. 7 compares the effectiveness between the three predictions under different modularity *Q*. Note that in real-world networks the relative errors increase with *Q*. In the 56 networks, in LHNs, and in LKNs, we note that the performances of the three predictions do not exhibit an obvious regularity versus the modularity, and in most cases the DMP method performs better than other two.

## Conclusions

In this study we have systematically examined the accuracies and relationships among the MFL, QMF, and DMP methods for predicting the epidemic threshold in the SIR model. To do this we have focused on a large number of artificial network simulations and on 56 real-world networks. We first analyzed the differences and correlations among the three theoretical epidemic threshold predictions. Generally speaking, the three predictions differ, and the epidemic threshold predicted by the DMP method is often larger than that predicted by the QMF method. In uncorrelated networks, the DMP and MFL methods produce the same epidemic threshold prediction, which is larger than the prediction produced by the QMF method. When applied to real-world networks, however, the relationships among the three predictions are still unclear.

We then checked the accuracies of the three predictive methods using uncorrelated configuration networks, and found that the MFL and DMP methods perform well, but that the QMF method does not. In the group of 56 real-world networks we found that the DMP method performs the best in most occasions, and that the epidemic threshold predicted by the MFL method is more accurate than the one predicted by the QMF method in most of the networks. In networks with an eigenvector localized on high *k*-core nodes, i.e., LKNs, the MFL method performs the best and the QMF method the worst, but in networks with an eigenvector localized on hubs, i.e., LHNs, the DMP method performs the best and the MFL method the worst.

Finally we measured the performances of the three methods versus the microscopic and mesoscale topologies in the 56 real-world networks, including degree-degree correlations *r*, clustering *c*, and modularity *Q*. For this purpose, we compute the average relative errors between theoretical thresholds and accurate thresholds for the networks in the interval (*x* − Δ*x*/2, *x* + Δ*x*/2), where *x* is *r*, *c*, and *Q*. The smaller value of the relative error indicates the better performance of the theory. In networks with negative degree-degree correlations, we found that the DMP method performs the best, and the QMF method performs than the MFL method. In the networks with positive degree-degree correlations, the MFL method is the most accurate, and the QMF method is the least. In networks with low clustering, the DMP method is the most accurate, and the MFL method is the least. In networks with high clustering, the MFL method is the most accurate, and the QMF method is the least. The relative accuracies of the three predictions versus the modularity are, unfortunately, irregular.

Predicting accurate epidemic thresholds in networks is profoundly significant in the field of spreading dynamics. Our results present a counterintuitive insight into the use of network information in theoretical methods, i.e., the performance level of a method is not only proportional to the topological information used, but also correlates with the dynamical correlations among the states of neighbor nodes. Our results expand our understanding of epidemic thresholds and provide ways of determining which method of theoretical prediction is best in a variety of given situations. Our results also indicate directions for further research into the development of more accurate theoretical methods of predicting epidemic thresholds. It should be noted that we just considered the SIR spreading dynamics with synchronous updating method, whether or not the results apply to the case with asynchronous updating method needs to be further studied. Some further investigations about the effects of network structural characteristics (e.g., degree-degree correlations) on the accuracy of the theoretical methods are still called for. For instance, one can study the effect of degree-degree correlations on the accuracy of the three theoretical methods by changing the degree-degree correlations^46^ of the configuration model gradually (see details in Supporting Information).

## Methods

### Predicting numerical threshold

To determine the theoretical epidemic threshold, we employ the estimators supplied by the MFL, QMF and DMP methods and use the relative variance *χ* to numerically determine the size-dependent epidemic threshold^47^,


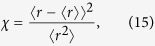


where *r* denotes the final epidemic size and 〈···〉 is the ensemble averaging. We use at least 10^5^ independent dynamic realizations on a network to calculate the average value of *χ*, which exhibits a maximum value at the epidemic threshold *λ*_*c*_. This numerical prediction *λ*_*c*_ obtained by observing *χ* we consider the accurate epidemic threshold^47^. The Supporting Information supplies illustrations of numerically locating the epidemic threshold by observing *χ*. There are also other ways of determining *λ*_*c*_, e.g., susceptibility^27^ and variability methods^48^.

## Additional Information

**How to cite this article**: Wang, W. *et al.* Predicting the epidemic threshold of the susceptible-infected-recovered model. *Sci. Rep.* **6**, 24676; doi: 10.1038/srep24676 (2016).

## Supplementary Material

Supplementary Information
