# Supplementary material for: Predicting the epidemic threshold of the susceptible-infected-recovered model
Source: Sci Rep. 2016 Apr 19;6:24676. doi: 10.1038/srep24676 (PMC4835734; doi:10.1038/srep24676)
Supplement: Supplementary Information [file srep24676-s1.pdf]

Supporting Information for 'Predicting the epidemic threshold of the susceptible-infected-recovered model'

Wei Wang,[1,2,3] Quan-Hui Liu,[1,2] Lin-Feng Zhong,[1,2] Ming Tang,[1,2] Hui Gao,[1,2] and H. Eugene Stanley[3]

[1]*Web Sciences Center, University of Electronic Science and Technology of China, Chengdu 610054, China*

[2]*Big data research center, University of Electronic Science and Technology of China, Chengdu 610054, China*

[3]*Center for Polymer Studies and Department of Physics, Boston University, Boston, Massachusetts 02215, USA*

(Dated: March 11, 2016)

I. SYNCHRONOUS UPDATING METHOD FOR SIR MODEL

We implement the synchronous updating method [1] to renew the states of nodes in the SIR model. Initially, a node u is randomly selected as seed (infected), while the remaining nodes are susceptible. Two queues Q_1 and Q_2 are used to store nodes which are infected in previous steps and current step, respectively. At time step $t = 0$, put the seed node u into the end of Q_1. When $t \geq 1$, the updating processes at every time step are executed as follows: (1) Set $t \rightarrow t+1$. (2) Every node in Q_1 tries to transmit the disease to each neighbor v. If node v is susceptible, v becomes infected with probability β, and node v is put into the end of queue Q_2; otherwise, nothing happens. (3) Every node u in Q_1 recovers with probability γ. If node u recovers, we move it from Q_1. (4) Add all nodes of Q_2 at the rear of Q_1, and delete all nodes in Q_2. Repeat steps (1)-(4) until all infected nodes become recovered. Finally, we count the fraction of nodes being in the recovered state, which is denoted as the epidemic size r.

II. NUMERICAL IDENTIFYING EPIDEMIC THRESHOLD

We use the relative variance χ to numerically determine the size-dependent critical value [2]

$$\chi = \frac{\langle r - \langle r \rangle \rangle^2}{\langle r^2 \rangle}, \tag{1}$$

where r denotes the final epidemic size and $\langle \cdots \rangle$ is the ensemble averaging. We use at least 10^5 independent dynamic realizations on a network to calculate the average value of χ, which exhibits a maximum value at the epidemic outbreak threshold λ_c. This numerical prediction λ_c by observing χ can be considered the accurate epidemic threshold.

Figure 1 shows how λ_c is located by observing χ in a scale-free network and a California network. In scale-free networks with degree exponent $\nu_D = 3.5$, we find that the MFL and DMP methods produce results close to the accurate epidemic threshold λ_c. In the California network, the epidemic threshold predicted by MFL method is closer to λ_c than the two other methods.

III. CORRELATED CONFIGURATION NETWORKS

We generate the correlated configuration networks according to the method in Ref. [3]. As shown in Fig. 2, the DMP method performs better than MFL and QMF methods.

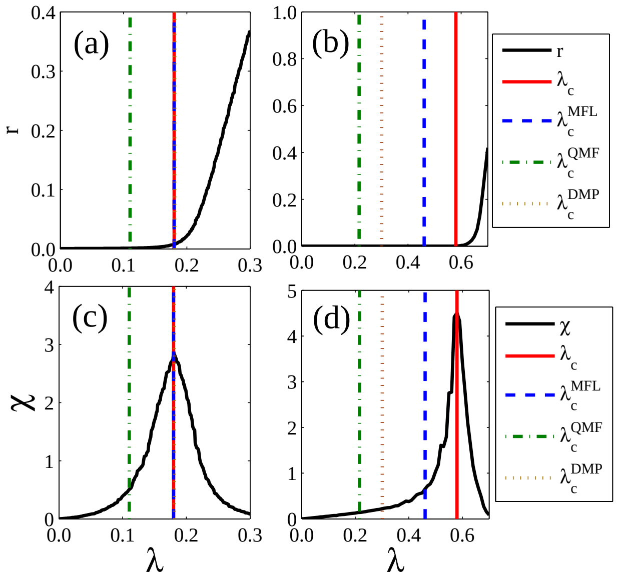

FIG. 1. (Color online) **Illustrations of the numerical identification of epidemic threshold.** The epidemic size r (a) and relative variance χ (c) versus the effective spreading probability λ on scale-free networks with degree exponent $\nu_D = 3.5$. The epidemic size r (b) and relative variance χ (d) versus λ for California network [22]. In (a) and (b), the solid black curves denote r and χ, respectively. The four vertical lines are the epidemic thresholds which are predicted by the numerical simulations λ_c (red solid lines), MFL method $\lambda_c^{\rm MFL}$ (blue dished lines), QMF method $\lambda_c^{\rm QMF}$ (green dish-dotted lines), and DMP method $\lambda_c^{\rm DMP}$ (purple dotted lines), respectively.

IV. REAL-WORLD NETWORKS

In this paper, we predict the SIR epidemic threshold for 56 real-networks, which are downloaded from website [4]. The 56 include social networks, coauthorship networks, metabolic networks, infrastructure networks, and citation networks. Table I displays their statistical characteristics in detail.

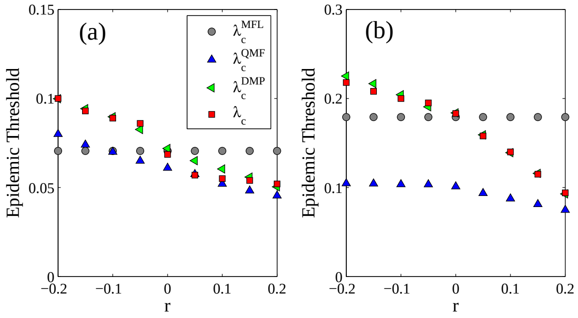

FIG. 2. (Color online) **Predicting epidemic thresholds for correlated configuration networks.** Theoretical predictions of $\lambda_c^{\rm MFL}$ (black circles), $\lambda_c^{\rm QMF}$ (blue up triangles), $\lambda_c^{\rm DMP}$ (green left triangles) and numerical predictions (red squares) as a function of degree-degree correlations r for degree exponent $\nu_D = 2.1$ (a) and $\nu_D = 3.5$ (b). The networks size is set to be $N = 8,000$.

[1] Schonfisch, B. & De Roos, A. Synchronous and asynchronous updating in cellular automata. Bio. Syst. **51**, 123 (1999).

[2] Chen, W., Schroder, M., & D'Söuza, M. R. Microtransition Cascades to Percolation. *Phys. Rev. Lett.* **112**, 155701 (2014).

[3] Van Mieghem, P., et al. Influence of assortativity and degree-preserving rewiring on the spectra of networks. Eur. Phys. J. B 76(4), 643-652 (2010).

[4] http://konect.uni- koblenz.de/networks/

[5] Leskovec, J., Kleinberg, J. & Faloutsos, C. Graph evolution: Densification and shrinking diameters. *ACM Trans. Knowledge Discovery from Data*. **1**(1), 1-40 (2007).

[6] Yang, J. & Leskovec, J. Defining and evaluating network communities based on ground-truth. *In Proc. ACM SIGKDD Workshop on Mining Data Semantics*. **3** (2012).

[7] Massa, P., Salvetti, M., & Tomasoni, D. Bowling alone and trust decline in social network sites. *In Proc. Int. Conf. Dependable, Autonomic and Secure Computing*. 658-663 (2009).

[8] McAuley, J. & Leskovec, J. Learning to discover social circles in ego networks. *In Advances in Neural Information Processing Systems*. 548-556 (2012).

[9] Choudhury, M. D., *et al.* How does the data sampling strategy impact the discovery of information diffusion in social media? *In ICWSM*. 34-41 (2010).

[10] McAuley, J. & Leskovec, J. Learning to discover social circles in ego networks. *In Advances in Neural Information Processing Systems*. 548-556 (2012).

[11] Cho, E., Myers, S. A. & Leskovec, J. Friendship and mobility: User movement in location-based social networks. *In Proc. Int. Conf. on Knowledge Discovery and Data Mining*. 1082-1090 (2011).

[12] Richardson, M., Agrawal, R. & Domingos, P. Trust management for the semantic web. *In The Semantic Web-ISWC*. 351-368 (2003).

[13] Mislove, A., *et al.* Measurement and analysis of online social networks. *In Proc. Internet Measurement Conf.* (2007).

[14] Viswanath, B., *et al.* On the evolution of user interaction in Facebook. *In Proc. Workshop on Online Social Networks*. 37-42 (2009).

[15] Cho, E., Myers, S. A. & Leskovec, J. Friendship and mobility: User movement in location-based social networks. *In Proc. Int. Conf. on Knowledge Discovery and Data Mining*. 1082-1090 (2011).

[16] Duch, J. & Arenas, A. Community detection in complex networks using extremal optimization. *Phys. Rev. E* **72**(2), 027104 (2005).

[17] Joshi-Tope, G., *et al.* Reactome: A knowledgebase of biological pathways. *Nucleic Acids Research*. **33**, 428-432 (2005).

[18] Ewing, R. M. *et al.* Large-scale mapping of human protein-protein interactions by mass spectrometry. *Molecular Systems Biology*. 3 (2007).

[19] Stelzl, U., *et al.* A human protein-protein interaction network: A resource for annotating the proteome. *Cell*. **122**, 957-968 (2005).

[20] Beuming, T., *et al.* PDZBase: A protein-protein interaction database for PDZ-domains. *Bioinformatics*. **21**(6), 827-828 (2005).

[21] Rual, J.-F., *et al.* Towards a proteome-scale map of the human protein-protein interaction network. *Nature*. **7062**, 1173-1178 (2005).

[22] Leskovec, J., *et al.* Statistical properties of community structure in large social and information networks. *In Proc. Int. World Wide Web Conf.* 695-704 (2008).

[23] Šubelj, L. & Bajec, M. Robust network community detection using balanced propagation. *Eur. Phys. J. B*. **81**(3), 353-362 (2011).

[24] Leskovec, J., *et al.* Community structure in large networks: Natural cluster sizes and the absence of large well-defined clusters. Internet Mathematics. **6**(1), 29-123 (2009).

[25] Opsahl, T., Agneessens, F. & Skvoretz, J. Node centrality in weighted networks: Generalizing degree and shortest paths. *Social Networks*. **3**(32), 245-251 (2010).

[26] Bollacker, K., Lawrence, S. & Giles, C. L. CiteSeer: An autonomous Web agent for automatic retrieval and identification of interesting publications. *In Proc. Int. Conf. on Autonomous Agents*. 116-123 (1998).

[27] Šubelj, L. & Bajec, M. Model of complex networks based on citation dynamics. I*n Proceedings of the WWW Workshop on*

TABLE I. Statistical characteristics and the theoretical epidemic thresholds of the 56 real-world networks. The statistical characteristics including the network size (N), number of edges (E), minimum degree (k_{min}), maximum degree (k_{max}), first ($\langle k\rangle$) and second moments ($\langle k^2\rangle$) of degree distribution, degree-degree correlations (c), clustering (c), modularity (Q), the inverse participation ratios of the adjacent matrix $IPR(\Lambda_A^1)$ and non-backtracking matrix $IPR(\Lambda_M^1)$). The theoretical epidemic thresholds are the MFL method (λ_c^{MFL}), the QMF method (λ_c^{QMF}) and DMP method (λ_c^{DMP}).

Category	Networks	Statistical Characteristics of Networks										Theoretical Epidemic Thresholds		
		N	E	k_{max}	$\langle k\rangle$	$\langle k^2\rangle$	r	c	Q	$IPR(\Lambda_A^1)$	$IPR(\Lambda_M^1)$	λ_c^{MFL}	λ_c^{QMF}	λ_c^{DMP}
Coauthorship	arXiv astro-ph [30]	17903	196972	504	22.004	1445.8	0.201	0.318	0.493	0.004	0.004	0.015	0.011	0.011
	arXiv hep-ph [30]	28045	3148414	4909	224.53	149810	0.033	0.28	0.408	0.0006	0.0006	0.002	0.001	0.001
	arXiv hep-th [30]	22721	2444642	8718	215.19	188080	−0.034	0.269	0.328	0.0008	0.0008	0.001	0.001	0.001
	DBLP co-authorship [6]	317080	1049866	343	6.622	144.01	0.267	0.306	0.734	0.008	0.008	0.048	0.009	0.009
Social	Advogato [7]	5042	39227	803	15.56	1284	−0.096	0.092	0.337	0.009	0.007	0.012	0.014	0.015
	Google+ [8]	23613	39182	2761	3.319	1251.7	−0.389	0.004	0.725	0.095	0.015	0.003	0.016	0.021
	Twitter (ICWSM) [9]	465017	833540	677	3.585	812.11	−0.878	0.0006	0.665	0.001	0.001	0.004	0.012	0.013
	Twitter lists [29]	22322	31823	238	2.851	112.05	−0.49	0.022	0.868	0.046	0.022	0.026	0.042	0.050
	Facebook (NIPS) [29]	2888	2981	769	2.0644	528.13	−0.668	0.0004	0.809	0.244	0.012	0.004	0.036	0.134
	Gowalla [11]	196591	950327	14730	9.668	2964	−0.029	0.023	0.621	0.018	0.005	0.003	0.006	0.006
	Epinions [12]	75877	405739	3044	10.695	1966.5	−0.041	0.066	0.386	0.002	0.002	0.005	0.005	0.006
	Hamsterster friendships [4]	1788	12476	272	13.955	635.61	−0.089	0.09	0.396	0.01	0.009	0.022	0.022	0.023
	Youtube friendship [6]	1134890	2987624	28754	5.265	2603.7	−0.037	0.006	0.632	0.048	0.005	0.002	0.005	0.005
	Hamsterster full [4]	2000	16098	273	16.098	704.71	0.023	0.23	0.45	0.009	0.008	0.023	0.02	0.021
	Youtube links [13]	1134885	2987468	28747	5.265	2601.1	−0.037	0.006	0.657	0.048	0.005	0.002	0.005	0.005
	Facebook Friendships [14]	63392	816831	1098	25.771	2268.9	0.177	0.148	0.506	0.001	0.001	0.011	0.008	0.008
	Brightkite [15]	56739	212945	1134	7.506	480.61	0.01	0.111	0.591	0.006	0.006	0.016	0.010	0.010
Metabolic	Caenorhabditis elegans [16]	453	2025	237	8.9404	358.49	−0.226	0.124	0.401	0.038	0.025	0.0256	0.038	0.043
	Reactome [17]	5973	145778	855	48.812	6995.1	0.241	0.606	0.719	0.004	0.004	0.007	0.005	0.005
	Human protein (Figeys) [18]	2217	6418	314	5.79	324.93	−0.332	0.008	0.472	0.027	0.017	0.018	0.032	0.036
	Human protein (Stelzl) [19]	1615	3106	95	3.846	65.648	-0.202	0.006	0.601	0.02	0.016	0.062	0.057	0.065
	PDZBase [20]	161	209	21	2.596	15.255	−0.466	0.003	0.755	0.1	0.05	0.205	0.171	0.248
	Human protein (Vidal) [21]	2783	6007	129	4.317	68.103	−0.137	0.035	0.615	0.029	0.012	0.068	0.063	0.076
Infrastructure	California [22]	1957027	2760388	12	2.821	8.9412	0.121	0.06	0.991	0.085	0.032	0.461	0.216	0.301
	Euroroad [23]	1039	1305	10	2.512	7.7536	0.09	0.035	0.862	0.0493	0.016	0.479	0.249	0.391
	Texas [24]	1351137	1879201	12	2.782	8.75	0.127	0.06	0.99	0.112	0.041	0.466	0.204	0.281
	Pennsylvania [24]	1087562	1541514	9	2.835	9.07	0.122	0.059	0.988	0.109	0.027	0.455	0.226	0.322
	Air traffic control [4]	1226	2408	34	3.928	28.899	−0.015	0.064	0.686	0.019	0.012	0.157	0.109	0.134
	OpenFlights [25]	2905	15645	242	10.771	601.45	0.049	0.255	0.581	0.01	0.009	0.018	0.016	0.016
Citation	arXiv hep-ph [30]	34401	420784	846	24.463	1553.4	−0.006	0.146	0.553	0.004	0.003	0.016	0.013	0.013
	arXiv hep-th [30]	27400	352021	2468	25.695	2733.8	−0.03	0.12	0.523	0.009	0.005	0.009	0.009	0.009
	CiteSeer [26]	365154	1721981	1739	9.432	456.97	−0.063	0.05	0.664	0.018	0.007	0.021	0.017	0.019
	Cora citation [27]	23166	89157	377	7.697	182.3	−0.055	0.117	0.683	0.01	0.008	0.044	0.032	0.034
	DBLP [28]	12495	49563	709	7.933	347.28	−0.046	0.062	0.538	0.028	0.011	0.023	0.023	0.026
Misc	Flickr [29]	105722	2316668	5425	43.826	15304	0.247	0.402	0.634	0.001	0.001	0.003	0.002	0.002
	Amazon (TWEB) [30]	403364	2443311	2752	12.115	370.15	−0.018	0.166	0.74	0.089	0.005	0.034	0.017	0.025
HumanSocial	Jazz musicians [31]	198	2742	100	27.697	1070.2	0.02	0.52	0.439	0.014	0.014	0.027	0.025	0.026
	Adolescent health [32]	2539	10455	27	8.236	86.414	0.251	0.142	0.597	0.022	0.017	0.105	0.076	0.084
	Physicians [33]	117	465	26	7.949	79.162	−0.084	0.175	0.372	0.025	0.018	0.112	0.099	0.114
Computer	Route views [30]	6474	12572	1458	3.884	640.08	−0.182	0.01	0.612	0.087	0.022	0.006	0.022	0.029
	CAIDA [30]	26475	53381	2628	4.033	1130.1	−0.195	0.007	0.639	0.024	0.01	0.004	0.014	0.017
	Gnutella [34]	62561	147878	95	4.728	54.86	−0.093	0.004	0.502	0.001	0.001	0.094	0.076	0.087
Communication	U. Rovira i Virgili [35]	1133	5451	71	9.622	179.82	0.078	0.166	0.511	0.01	0.008	0.057	0.048	0.052
	EU institution [30]	224832	339925	7636	3.024	1716.5	−0.189	0.004	0.729	0.003	0.003	0.002	0.01	0.01
Lexical	WordNet [36]	145145	656230	1008	9.042	503.2	−0.063	0.096	0.704	0.028	0.024	0.018	0.014	0.015
	King James [4]	1707	9059	364	10.614	441.85	−0.052	0.162	0.461	0.023	0.017	0.025	0.025	0.027
	David Copperfield [37]	112	425	49	7.589	104.54	−0.129	0.157	0.295	0.047	0.034	0.078	0.076	0.087
Hyperlink	Google.com internal [38]	15763	148585	11401	18.852	16998	−0.122	0.013	0.48	0.043	0.021	0.001	0.006	0.006
	Notre Dame [39]	325729	1090108	10721	6.693	1878.7	−0.053	0.088	0.927	0.023	0.008	0.004	0.005	0.006
	Stanford [24]	255265	1941926	38625	15.215	30898	−0.116	0.009	0.892	0.024	0.022	0.0005	0.002	0.002
Trophic	Little Rock Lake [40]	183	2434	105	26.601	1140.9	−0.267	0.332	0.345	0.015	0.014	0.024	0.024	0.025
	Florida ecosystem dry [41]	128	2106	110	32.906	1332.7	−0.104	0.314	0.146	0.015	0.014	0.025	0.025	0.026
	Florida ecosystem wet [41]	128	2075	110	32.422	1300.3	−0.112	0.312	0.137	0.015	0.014	0.026	0.025	0.026
Animal Animal	Dolphins [42]	62	159	12	5.129	34.903	−0.044	0.309	0.495	0.053	0.044	0.172	0.139	0.167
OnlineContact	Pretty Good Privacy [43]	10680	24316	205	4.554	85.976	0.238	0.378	0.847	0.017	0.016	0.056	0.024	0.024
Software	Linux [4]	30817	213208	9338	13.837	11798	−0.175	0.003	0.427	0.026	0.015	0.001	0.006	0.006

Large Scale Network Analysis. 527-530 (2013).

[28] Ley, M. The DBLP computer science bibliography: Evolution, research issues, perspectives. *In Proc. Int. Symposium on String Processing and Information Retrieval*. 1-10 (2002).

[29] McAuley, J. & Leskovec, J. Learning to discover social circles in ego networks. *In Advances in Neural Information Processing Systems*. 548-556 (2012).

[30] Leskovec, J., Adamic, L. A. & Huberman, B. A. The dynamics of viral marketing. *ACM Trans. on the Web*. **1**(1) (2007).

[31] Gleiser, P. M. & Danon, L. Community structure in jazz. *Advances in Complex Systems*. **6**(4), 565-573 (2003).

[32] Moody, J. Peer influence groups: Identifying dense clusters in large networks. *Social Networks*. **23**(4), 261-283 (2001).

[33] Coleman, J., Katz, E. & Menzel, H. The diffusion of an innovation among physicians. *Sociometry*. 253-270 (1957).

[34] Ripeanu, M., Foster, I. & Iamnitchi, A. Mapping the Gnutella network: Properties of large-scale peer-to-peer systems and implications for system design. *IEEE Internet Computing J.* 6 (2002).

[35] Guimerà, R., *et al*. Self-similar community structure in a network of human interactions. *Phys. Rev. E* **68**(6), 065103 (2003).

[36] Fellbaum, C. *WordNet: an Electronic Lexical Database* (MIT Press, 1998).

[37] Newman, M. E. J. Finding community structure in networks using the eigenvectors of matrices. *Phys. Rev. E* **74**(3), 036104 (2006).

[38] Palla, G., *et al*. Directed network modules. *New J. Phys.* **9**(6), 186 (2007).

[39] Albert, R., Jeong, H. & Barabási, A.-L. Internet: Diameter of the world-wide web. *Nature*. **401**(6749), 130-131 (1999).

[40] Martinez, N. D., *et al*. Artifacts or attributes? effects of resolution on the Little Rock Lake food web. *Ecological Monographs*. **61**, 367-392 (1991).

[41] Ulanowicz, R. E., *et al*. Annual Report to the United States Geological Service Biological Resources Division Ref. No.[UMCES] CBL 00-0176, Chesapeake Biological Laboratory, University of Maryland (2000).

[42] Lusseau, D., *et al*. The bottlenose dolphin community of Doubtful Sound features a large proportion of long-lasting associations. *Behavioral Ecology and Sociobiology*. **54**, 396-405 (2003).

[43] Boguñá, M., *et al*. Models of social networks based on social distance attachment. *Phys. Rev. E* **70**(5), 056122 (2004).
